# Etiological profile of nephrotic syndrome in Kashmir

**DOI:** 10.4103/0971-4065.41281

**Published:** 2008-01

**Authors:** A. R. Reshi, M. A. Bhat, M. S. Najar, K. A. Banday, M. A. Naik, D. P. Singh, F. Wani

**Affiliations:** Department of Nephrology, SKIMS, Soura, Srinagar, Jammu and Kashmir, India

**Keywords:** Minimal change disease, nephrotic syndrome, focal segmental glomerulosclerosis, end stage kidney disease

## Abstract

Our study aimed to obtain a comprehensive insight into the etiology of nephrotic syndrome in our patient population. We analyzed medical records of 290 patients with diagnosis of nephrotic syndrome as defined by International Study of Kidney Disease in Children (ISKDC), between January 1987 and December 2000, at the Sher-I-Kashmir Institute of Medical Sciences, Soura, Srinagar. Primary glomerular disease was found to be the most prevalent, accounting for 91.73% of all glomerular diseases. Among primary glomerular diseases, minimal change disease (MCD) was the most common histological lesion (43.79%). Most patients presented within 3 months duration (61.4%) and the most common symptom was puffiness of face (98.45%) followed by pedal edema (91%). Focal segmental glomerulosclerosis (FSGS) was the second most common lesion (16.89%) followed by membranous glomerulonephritis (GN) (13.4%) and membranoproliferative GN (11.72%). Amongst secondary glomerular diseases, diabetes mellitus was the most prevalent (4.48%), followed by lupus nephritis (3.1%). In conclusion, primary glomerular diseases constituted the most common group encountered and the prevalence of MCD was quite high with males, children and young adults. FSGS was associated with a high prevalence of end-stage renal disease (ESRD; 26.53%), hypertension (71.42%) and hematuria (81.63%).

## Introduction

Nephrotic syndrome (NS) is defined by massive continued losses of urinary proteins, resulting in hypoalbuminemia and edema. These are associated with complications such as increased susceptibility to infections, thromboembolism, altered lipid and carbohydrate metabolism and losses in binding proteins in the urine.^1^

Despite considerable advances in health care, glomerular disease constitutes one of the leading causes of renal failure resulting in considerable morbidity and mortality. The patterns of the glomerular diseases are different in different countries and are changing with time within the same country, probably due to better infection control, changes in environmental pollution, increased awareness of the disease and changes in life expectancy. For instance, immunoglobulin A nephropathy (IgAN) is common in the Northwest regions of Italy,^2^ the far East, and eastern Europe,^3^,^4^ while focal and segmental glomerulosclerosis (FSGS) appears most prevalent in the United States of America.^5^ Similarly, FSGS is the most common lesion reported from Saudi Arabia.^6^ The prevalence of FSGS appears to be increasing as reported from New York where the prevalence increased from 2.5% to 18.7% over a 20-year period.^7^ The prevalence of FSGS has marked racial differences being more common in African American population presenting with nephrotic syndrome.^8^–^10^ Recent studies have shown the association of glomerulonephritis (GN) with the hepatitis C virus (HCV).^11^ Breen *et al.*, have found that HCV may play a role in the development of FSGS.^12^

The prevalence of membranous GN (MGN) has not changed in the last 20 years and remains the main cause of the nephrotic syndrome in European adults.^7^,^8^ In Italy, Japan, China, Hong Kong, Singapore and Taiwan, IgAN was the most common of all primary GNs followed by MGN and FSGS,^2^,^3^,^13^–^15^ while MGN was the most common lesion encountered in China and Indonesia.^16^

In Thailand, IgAN followed by FSGS and membranous nephropathy represent the most common cause of nephrotic syndrome.^17^ In Iraq, FSGS followed by mesangial glomerular nephritis and minimal change disease (MCD) represent the most common primary glomerular diseases.^18^

Secondary glomerular disease accounted for 54% of all glomerular diseases in Jamaica^19^ and hepatitis B was associated with 80% of glomerular diseases in Zimbabwe.^20^ Lupus nephritis accounted for 20.4% in Hawaii^21^ and was three times more common in Africans than Europeans in the USA.^22^

Moreover, in India, the pattern varies according to the demographic location, mesangioproliferative GN represents the most common cause of nephrotic syndrome from South India,^23^ whereas primary IgAN is more common in young adults in the second to third decade of life from western India^24^ and MCD dominates northern India.^25^,^26^

This study was conducted at Sher-I-Kashmir Institute of Medical Sciences (SKIMS), Soura, a tertiary care hospital with a heterogeneous population representing patients from almost all parts of the Kashmir valley.

## Materials and Methods

We analyzed the medical records of 290 consecutive patients with renal biopsy diagnosed with GN at the SKIMS, Soura, between January 1987 and December 2000. The study parameters included age, sex, nationality, presenting symptoms and blood pressure of the patients, complete blood picture, urine analysis and microscopy, 24-h urinary protein excretion, creatinine clearance, serum electrolytes, serum urea and creatinine levels, serology and immunological studies, serological markers for hepatitis B and C, antibody against the human immunodeficiency virus (HIV), ultrasound, renal histopathology, treatment provided and outcome. Renal tissue was obtained by percutaneous biopsy using a Tru-Cut needle and the tissue was processed for light microscopy (LM) and immunofluoroscence (IF). Only biopsy specimens containing four or more glomeruli were considered appropriate. In all cases, a minimum of 20 sections were obtained and stained with hematoxylin-eosin, periodic acid-Schiff (PAS), trichrome and Jones' Silver stain. The steroid sensitive nephrotic syndrome was diagnosed in children with proteinuria, without hypertension and with benign urinary sediment without performing renal biopsy; these children had responded to the usual doses of steroids (prednisolone) within 8 weeks of initiating the treatment. Renal biopsy was performed only in patients who were above 12 years of age and those below 12 years who were steroid resistant.

## Results

The total number of patients enrolled during the study period was 290. Males outnumbered females with a male to female ratio of 2:1. Age ranged from 1 to 72 years with a mean of 25.47 ± 13.77 years. A total of 173 patients were in the age group 18-65 years (group 2), followed by 87 in the pediatric group (1-18 years [group 1]) and 31 in the elderly group (>65 years [group 3]). One hundred and seventy-seven (61%) patients presented with symptoms suggestive of nephrotic syndrome for 0-3 months. The most common symptoms were puffiness of face (99%) and pedal edema (91%), oliguria (42.7%), constitutional symptoms (22%), hypertension (20.6%) and dyspnea and orthopnea (14.4%). Majority of the patients were normotensive (79.4%), whereas mild (10.6%), moderate (5%) and severe (5%) hypertension was noted in the rest.

Primary GN was observed in 266 patients (91.73%). MCD was the predominant lesion, observed in 127 cases (43.79%). The other lesions encountered included focal segmental glomerulosclerosis (n = 49, 16.89%), membranous GN (n = 39, 13.4%), mesangiocapillary GN (n = 34, 11.72%), diffuse proliferative GN (n = 7, 2.41%), mesangioproliferative GN (n = 6, 2.06%) and IgAN (n = 4, 1.37%).

Secondary glomerular disease (SGD) was found in 24 cases (8.27%); in 13 (4.48%) of these, diabetes mellitus was the cause. Systemic lupus erythematosus (SLE) was present in 9 cases (3.1%) and glomerular disease secondary to amyloidosis was present in 2 (0.68%).

MCD was the most common cause in adult (33.52%) and pediatric (73.25%) groups followed by FSGS (19.07% in adult group and 9.3% in pediatric group). Membranous GN was common in the elderly group (32.25%) followed by FSGS (25.8%). MCD was predominantly observed in males (62.99%) with puffiness of face as the most common symptom (99%) and in 61.4% patient, the duration of presentation was less than three months. Hematuria was observed in 7.86% of patients; hypertension, in 3.93% and renal impairment, in 2 (1.57%). On the contrary, FSGS had higher incidence of hematuria (81.63%), hypertension (71.42%) and renal insufficiency (26.53%).

## Discussion

In the present study, primary glomerular disease was the predominant cause of nephrotic syndrome and accounted for 91.73% of all biopsies. MCD was the most prevalent disorder and constituted 43.79% of total renal biopsies and 47.74% of all primary glomerular diseases. This is in accordance with previously published studies from India.^25^–^27^ Membranous GN was the most common cause in the elderly group as observed by others.^28^,^29^ FSGS was the second most common cause. This is in accordance with observations made by others^29^ and some reported it as the most common cause in children above 8 years of age.^30^ The incidence of primary and secondary glomerular diseases is similar to that found in a study conducted in Iraq.^18^

The prevalence of MCD also varies within India, being less than 12% in Vellore in the Southern part of the country^23^ to approximately 33% in Haryana.^26^ The reported prevalence of MCD in other countries is less than 17% in Thailand and Iraq.^17^,^18^

MCD was the leading cause of nephrotic syndrome in pediatric and adult groups as observed in 43.79% of our study patients. Although FSGS is reported as the most common cause of nephrotic syndrome from Southeast Asia, MCD has been reported as the leading cause from Indonesia and Vietnam.^31^ Contrary to far Eastern countries such as China, Hong Kong and Taiwan, where IgAN is the most common glomerular lesion accounting for 50% of the total glomerular diseases,^3^,^13^ IgAN was observed in only 1.37% of all adult patients in our study. IgAN was more frequent in younger patients and presented most commonly with hematuria.

The prevalence of amyloidosis in our study group was less than 1% despite the high prevalence of rheumatoid arthritis, tuberculosis and other chronic conditions that may be associated with amyloidosis.

Amongst secondary GN, diabetes mellitus was the most prevalent lesion observed in our study, although the overall prevalence is lower than that observed in other parts of the country. This can be explained by the late presentation to a tertiary care hospital and resentment toward renal biopsy. A similar high prevalence of the disease has been reported from AIIMS, New Delhi,^25^ while a high prevalence of amyloidosis as a cause of secondary glomerular disease has been reported from PGI, Chandigarh.^32^ Regarding the outcome of the different glomerular diseases, FSGS had a fairly high progression rate to end-stage renal disease (ESRD) among primary glomerular disease, while diabetes had a high incidence of ESRD among secondary glomerular disease.

In conclusion, MCD was the most common primary glomerular disease observed in the present study and is more common in males, children and adults. FSGS, the second most common cause of nephrotic syndrome in all age groups, is associated with a high incidence of hematuria, hypertension and renal impairment. Since the etiology of FSGS is unclear, it is necessary to explore the possible association of factors, which could shed some light on this problem. The clinicopathological spectrum of nephrotic syndrome in Kashmir valley is similar to that observed in northern India.
